# Increased chemotactic migration and growth in heparanase-overexpressing human U251n glioma cells

**DOI:** 10.1186/1756-9966-27-23

**Published:** 2008-07-22

**Authors:** Xin Hong, Feng Jiang, Steven N Kalkanis, Zheng Gang Zhang, Xuepeng Zhang, Xuguang Zheng, Hao Jiang, Tom Mikkelsen, Michael Chopp

**Affiliations:** 1 Department of Neurosurgery, Henry Ford Health Science Center, Detroit, MI, USA; 2 Department of Neurology, Henry Ford Health Science Center, Detroit, MI, USA; 3 Physics Department, Oakland University, Rochester, MI, USA

## Abstract

Heparanase is an endoglycosidase that degrades heparan sulfate, the main polysaccharide constituent of the extracellular matrix (ECM) and basement membrane. Expression of the heparanase gene is associated with the invasion and metastatic potential of a variety of tumor-derived cell types. However, the roles of heparanase in the regulation of gene expression and the subsequent cell function changes other than invasion are not clear. In the current study, we overexpressed the human heparanase gene in a human U251n glioma cell line. We found that heparanase-overexpression significantly increased cell invasion, proliferation, anchorage-independent colony formation and chemotactic migration towards fetal bovine serum (FBS)-supplied medium and stromal cell-derived factor-1 (SDF-1). These phenotypic appearances were accompanied by enhanced protein kinase B (AKT) phosphorylation. Focal adhesion kinase (FAK) and extracellular signal-regulated kinase 1 (ERK1) signaling were not altered by heparanase-overexpression. These results indicate that heparanase has pleiotropic effects on tumor cells.

## Introduction

Tumor cell invasion and metastatic spread depend on the ability of cancer cells to invade tissue barriers by degrading extracellular matrix (ECM) and basement membrane structures [1,2]. The primary components of the basement membrane and ECM are structural proteins, such as collagen IV, laminin, fibronectin, and heparan sulfate proteoglycans (HSPGs). Heparan sulfate (HS) is a glycosaminoglycon (GAG) chain present in HSPGs [3]. HS chains interact through specific attachment sites with the main protein components of basement membrane and ECM.

Heparanase is a mammalian endo-β-D-glucuronidase responsible for HS degradation [4-7]. Heparanase activity may therefore play an important role in fundamental biological processes associated with ECM remodeling and cell invasion [8,9]. Increased expression of heparanase mRNA and protein has been reported in a variety of metastatic cell lines and human tumor tissues, whereas adjacent normal-looking tissue does not exhibit detectable levels of heparanase [10-15]. Moreover, increased heparanase mRNA expression correlates with reduced postoperative survival of cancer patients [12,15,16]. Overexpression of heparanase cDNA in tumor cells with low metastatic potential confers a high metastatic potential after injection of these cells into the experimental animals [4]. Heparanase has also been shown to elicit an angiogenic response by releasing heparan sulfate-bound angiogenic factors sequestered in the ECM, such as vascular endothelial growth factor (VEGF) and basic fibroblast growth factor (bFGF) [17,18].

Recently, it was reported that heparanase is translocated into the cell nucleus where it may degrade the nuclear heparan sulfate and thereby affect nuclear functions (i.e. regulation of gene expression and signal transduction) that are thought to be regulated by heparan sulfate [19]. Thus, the function of heparanase seems not to be only limited to degrading extracellular matrix. Cellular function may be affected by heparanase through regulating gene expression. There are accumulating evidences that certain signal transduction cascades are altered under heparanase stimulation [20-22]. However, genes and cellular functions involved in heparanase regulation, other than cell invasion, is still unclear. In the present study, we demonstrate that heparanase overexpression in stably transfected human U251n glioma cells results in a marked increase of cell chemotactic migration toward fetal bovine serum (FBS)-supplied medium and stromal cell-derived factor-1 (SDF-1). Cell proliferation and anchorage-independent growth are also increased in heparanase-overexpressing cells. These phenotypic changes are combined with elevated protein kinase B (AKT) phosphorylation.

## Materials and methods

### Antibodies and reagents

The following antibodies were purchased from Santa Cruz Biotechnology: anti-heparanase (H-80, sc-25825), anti-phospho-FAK and anti-actin. Antibodies for ERK1, phospho-ERK1, AKT, phospho-AKT (ser473), phosphor-GSK3β (ser9) and phosphor-eNOS (ser1177) were obtained from Cell Signaling Technology (Beverly, MA). Other antibodies included anti-FAK (44624G) (Biosource, Camarillo, CA) and integrin β1 (AB1952) (Chemicon, Temecula, CA). Growth factor reduced matrigel was purchased from Becton Dickinson (San Diego, CA). Heparin (H3149), dimethyl formamide (D4551) and 3-(4,5-dimethylthiazol-2-yl)-2,5-diphenyltetrazolium bromide (MTT) (M2128) were the products of Sigma (St. Louis, MO).

### Cell culture and transfection

U251n and U87 glioma cells originally were obtained from the American Type Culture Collection (ATCC). Cells were cultured at 37°C with 5% CO_2 _and maintained in DMEM containing 10% (v/v) fetal bovine serum (FBS), 4 mM glutamine, 100 IU/ml penicillin, 100 μg/ml streptomycin, and 1% nonessential amino acid (Invitrogen). Primary cultured glioma tumor cells (HF2303) were obtained from Hermelin Brain Tumor Center, Henry Ford Hospital (Detroit, MI, USA) with written consent in accordance with institutional guidelines. For stable transfection, U251n cells were transfected with the full-length human heparanase cDNA (kindly provided by Dr. Ian N. Hampson, University of Manchester, United Kingdom) or a control pcDNA3 vector, using the Lipofactamine 2000 reagent (Life Technologies). Cells were selected with G418 (800 μg/ml) for 3 weeks, expanded, pooled and further selected for high heparanase-expressing cells, as evaluated by real-time reverse transcription-PCR. The pool with the highest heparanase expression levels was labeled as "U51n-hpa" throughout the manuscript, whereas, the parental pcDNA3 transfected cells was referred to as "U251n-pc". U251n cells were also transfected with pcDNA3-Flag-HA-AKT1 (Addgene Inc. Cambridge, MA) for 48 hours. AKT overexpressing U251n cells (U251n-AKT) will be used as control for Western blot.

### Heparanase enzyme activity assay

Heparanase activities were assayed in cell lysates by using a heparan-degrading enzyme assay kit (TaKaRa Bio Inc.). One million cells were lysed with 1 ml extraction buffer, centrifuged at 10,000 × g for 5 min at 4°C, and then supernatants were collected as samples. Heparanase activities in all samples were interpolated from a standard curve performed by using an unlabeled HS as a standard substitute. The absorbance was read by a microplate reader (Multiskan MCC/340, Labsystems, Finland) at the wavelength of 450 nm.

### Real-time RT-PCR

Total RNAs were extracted using a RNeasy mini kit with DNase digestion (Qiagen, Santa Clarita, CA). Two step real-time PCR was performed as described previously [23]. Housekeeping gene TATA box binding protein (TBP) was used for each RNA sample as control. The mRNA expression was expressed as the fold change related to TBP mRNA expression. Primers included 5'-TGA TCT TGA CCA GAA TAC CAT CGA-3' and 5'-GGC TTG CGA GGG AAG AAG TT-3' for MMP-2 [GenBank: NM_004530], 5'-GAC AAG CTC TTC GGC TTC TG-3' and 5'-TCG CTG GTA CAG GTC GAG T-3' for MMP-9 [Gene bank:: NM_004994], 5'-CCT TGC TAT CCG ACA CCT TT-3' and 5'-CAC CAC TTC TAT TCC CAT TCG-3' for heparanase [GenBank: NM_006665], 5'-TGC ACA GGA GCC AAG AGT GAA-3' and 5'-CAC ATC ACA GCT CCC CAC CA-3' for TBP [GeneBank: NM_003194]. Specificity of the produced amplification product was confirmed by examination of dissociation reaction plots. A distinct single peak indicated that a single DNA sequence was amplified during RT-PCR. Each sample was tested in triplicate and samples obtained from three independent experiments were used for analysis of relative gene expression.

### Western blot analysis

Cell cultures were washed twice with ice-cold PBS, and scraped in lysis buffer (50 mM Tris pH 7.4, 250 mM NaCl, 5 mM EDTA, 1% NP40, 0.1% SDS, 0.5% sodium deoxycholate, 1 mM phenylmethylsulphonyl fluoride) containing 1% protease inhibitor cocktail (Calbiochem). Lysates were obtained by centrifugation at 13,000 rpm for 10 min at 4°C and protein concentration was determined using the BCA protein assay kit (Pierce, Rockford, IL). Twenty to forty μg of total protein were subjected to SDS-PAGE, transferred to polyvinylidene fluoride (PVDF) membrane, and probed with various primary antibodies, followed by HRP-conjugated secondary antibodies. Specific proteins were detected by enhanced chemiluminescence (Pierce). The experiments were repeated in triplicate. β-actin was used as the internal protein control.

### Matrigel invasion assay

Invasion of cells through matrix membrane was determined using 24-well BD invasion chambers (8.0-μm pore size with polycarbonate membrane; BD Biosciences, Cowley, United Kingdom) in accordance with the manufacturer's instructions with the following modifications. The cells were detached by using 2 mM EDTA and 5 × 10^4 ^cells were placed into the upper compartment of the invasion plates in duplicate in a 0.5 ml serum-free volume. Subsequently, the lower compartment was filled with 750 μl of 10%FBS medium. After 22 h of incubation at 37°C with 5% CO_2_, cells remaining on the upper membrane surface were removed with a cotton swab. Cells on the lower surface of the membrane were stained with CellTracker Green (Molecular Probes, Eugene, OR) and fixed in methanol. The invasive cells on the lower surface of membrane were counted under the fluorescence microscope at × 4 magnification. Each experiment was repeated three times.

### Zymographic analysis of matrix metalloproteinase

Total cell proteins were prepared in the same way as for Western blot analysis. Twenty micrograms of each lysate or 15 μl of cell culture supernatant were mixed with non-reducing electrophoresis loading buffer and subjected to electrophoresis on an 8% SDS-PAGE copolymerized with gelatin (1 mg/ml). After electrophoresis, gels were washed with 2.5% Triton X-100 for 1 h (3 times, 20 min each) and incubated for 24 h in enzyme assay buffer (25 mM Tris, pH 7.5, 5 mM CaCl_2_, 0.9% NaCl, and 0.05% Na_3_N) for the development of enzyme activity bands. After incubation, the gels were stained with 0.25% coomassie brilliant blue R-250 and destained in 10% methanol with 5% acetic acid. The gelatinolytic activities were detected as transparent bands against the blue background of the coomassie brilliant blue-stained gelatin. The experiment was repeated three times. Human MMP-2 and MMP-9 gelatinase zymography standard (Chemicon) were used as markers.

### Chemotactic Migration Assay

A Chemicon QCM™ 96-well migration assay kit was used to study cell chemotactic migration. Cells were detached by 2 mM EDTA/PBS and resuspended with serum-free medium. Approximately 5 × 10^4 ^cells were seeded in the upper chamber of the plate. Medium with 10%FBS or 100 ng/ml SDF-1 was added to the lower chamber as a chemoattractant, and serum-free medium was also used as control. After 6 h incubation, cells migrated through the 8 μm pore size membrane, were detached, and treated with lysis buffer/dye solution supplied by the kit. Aliquot mixes were read using fluorescence reader with 485/525 nm filter set. Cell numbers were correlated with the optical density values and expressed as Relative Fluorescence Unit (RFU). The experiments were repeated three times with at least four duplicate of each treatment.

### Cell proliferation assays

MTT assay was used to determine the cell proliferation. U251n-pc and U251n-hpa cells were initially grown to confluence before seeding in a 96-well plate at the density of 4 × 10^3 ^cells/well. Viable cells were determined by adding 0.5 mg/ml MTT into each well and incubated for additional 2 h. The cells were then solubilized in 200 μl detergent (50% dimethyl formamide and 10%SDS). The absorption was determined at the wavelength of 540 nm. Observed optical density is directly correlated with the cell numbers. The experiments were repeated three times with duplicates.

### Colony formation in soft agar

Three ml of DMEM containing 0.5% Low Melt agarose (Bio-Rad, Hercules, CA) and 10% FCS was poured into a 6-well plate. The layer was covered with cell suspension (1 × 10^4 ^cells) in 1.5 ml of DMEM containing 0.3% Low Melt agarose and 10% FCS, and the dish was covered with 2 ml of DMEM containing 10% FCS. Cells were seeded in triplicate and medium was changed every 3 days. After 3 weeks, colonies were visualized by MTT staining (0.5 mg/ml) for 4 h and counted under a microscope. The experiment was repeated three times.

### Statistics

Data are presented as mean ± SD. Statistical significance was analyzed by one-way ANOVA. The value of *P *< 0.05 was considered statistically significant.

## Results

### Heparanase increases U251n cell invasion

**Figure 1:**
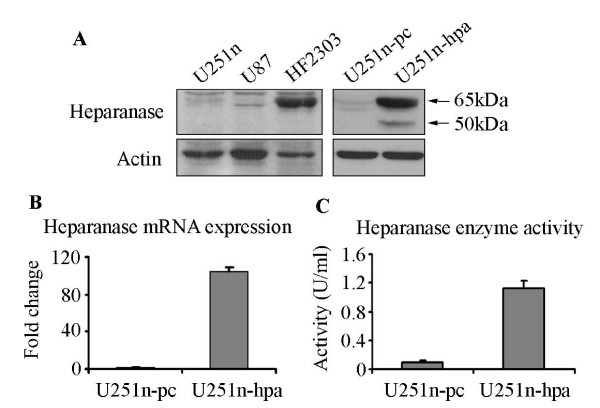
**Overexpression of heparanase in U251n cells**. (A) Heparanase expression of U251n, U87 and primary cultured glioma cells (HF2303) were detected by Western blot. Both U251n and U87 exhibited baseline level of heparanase expression. Latent form of heparanase was detected in HF2303 cells. Increased heparanase expression, both the latent (65 kDa) and active (50 kDa) forms, was observed in cell lysate derived from U251n-hpa cells, as compared to U251n-pc control cells. (B) U251n-hpa cells expressed about 100 times more mRNA than U251n-pc cells. (C) Increased heparanase activity was detected in U251n-hpa cells.

**Figure 2:**
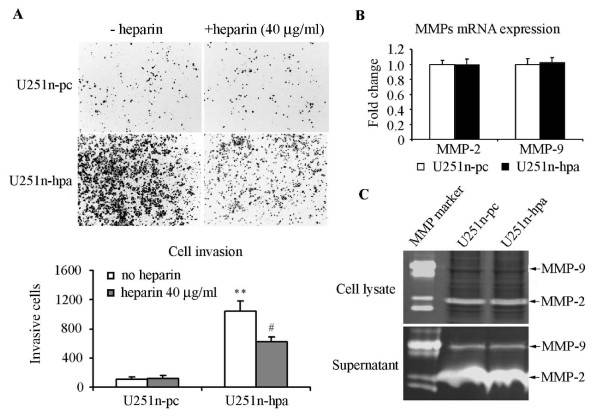
**Heparanase overexpression increases cell invasion**. (A) Cell invasion of U251n-hpa was significantly increased as compared to U251n-pc cells, measured by BD invasion assay. Increased cell invasion was greatly inhibited by 40 μg/ml heparin. **, *P *< 0.01, compared with U251n-pc cells. #, *P *< 0.01, compared with U251n-hpa group of no heparin treatment. (B) mRNA levels of MMP-2 and MMP-9 showed no significant difference between U251n-pc and U251n-hpa cells. (C) No significant differences were detected in MMP-2 and MMP-9 activity between U251n-hpa cells and U251n-pc cells as measured by zymography assay.

To understand the role of heparanase on the regulation of cell function, the full length human heparanase gene was stably transfected into U251n cells. Western blot analysis using cell lysates from heparanase-overexpressing U251n-hpa cells showed a great increase of protein expression of both latent (65 kDa) and active (50 kDa) forms of heparanase. Heparanase expression was confirmed by analyzing U87 and HF2303 cells (Figure 1A). U251n-hpa cells exhibited a 100 fold increase of mRNA expression (Figure 1B) and a 12-fold increase of heparanase activity in cell lysates compared to U251n-pc cells (Figure 1C). Heparanase cleaves HS residues, participates in ECM degradation, and thereby may facilitate tumor cell invasion [4]. Using the BD matrigel invasion assay, we evaluated the invasion ability of U251n cells. Matrigel, a reconstituted basement membrane containing HSPG, was used as a relevant barrier. As expected, the heparanase-overexpressing U251n-hpa cells exhibited a 10-fold increase in cell invasion as compared to control U251n-pc cells, and the increased cell invasion was significantly blocked by the heparanase inhibitor heparin at a concentration of 40 μg/ml (*P *< 0.01) (Figure 2A). In order to distinguish the role of heparanase on cell invasion, MMP-2 and MMP-9, members of the MMP family that is also involved in glioma invasion [24], was measured using zymography assay and real-time PCR in both heparanase-overexpressing and control cells. No significant changes were observed in both enzyme activity and mRNA expression levels between U251n-pc and U251n-hpa cells (Figure 2B and 2C).

### Heparanase enhances U251n cell chemotactic migration

**Figure 3:**
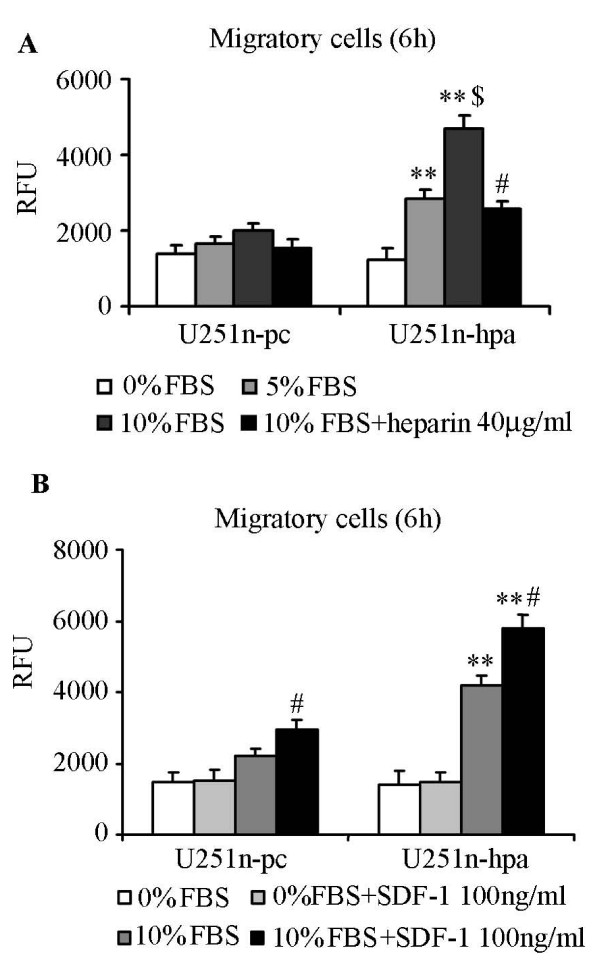
**Overexpression of heparanase increases U251n cell chemotactic migration**. (A) Heparanase-overexressing U251n-hpa cells showed increased migration towards FBS dose-dependent manner (5% and 10% FBS), and increased migration was significantly blocked by 40 μg/ml heparin. **, *P *< 0.01, compared with the same treatment of U251n-pc cells. $, *P *< 0.01, compared with 5% FBS group of U251n-hpa. #, P < 0.01, compared with 10% FBS group of U251n-hpa. (B) SDF-1 significantly increased cell chomatactic migration in the presence of 10% FBS medium. Increased migration was not observed when SDF-1 was added in serum free medium. **, *P *< 0.01, compared with the same treatment of U251n-pc cells. #, *P *< 0.01, compared with 10%FBS group of each cell line.

Apart from the traditional function of heparanase on cell invasion, cell migration towards chemoattractants was detected. Our results showed that migration of U251n-hpa cells was significantly increased towards DMEM medium supplied with 5% and 10% FBS as compared to U251n-pc cells (*P *< 0.01), while cell migration was significantly blocked by 40 μg/ml heparin (Figure 3A) (*P *< 0.01). These results indicate that increased migration is heparanase-induced cellular function change which is independent of ECM degradation. Since SDF-1 and its receptor CXCR4 (CXC chemokine receptor) system was reported to be correlated with cancer cell migration [25-27], SDF-1 was also tested as a chemoattractant. More cells were migrated when SDF-1 was added into the 10% FBS medium than 10% FBS medium alone (*P *< 0.01), and total number of migrating cells of U251n-hpa group was significantly higher than that of U251n-pc group (*P *< 0.01). The number of migrating cells was not increased when SDF-1 was added into 0% FBS medium (Figure 3B), indicating that SDF-1 induced migration needs the activation of other cellular signals.

### Heparanase overexpression increases U251n cell growth

**Figure 4:**
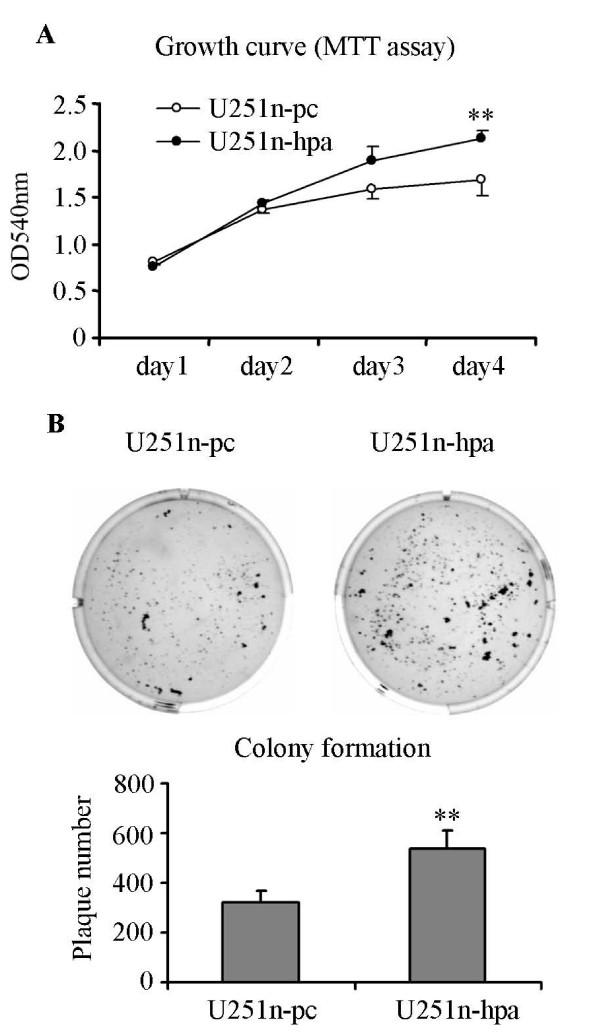
**Overexpression of heparanase enhances U251n cell growth**. (A) U251n-hpa showed increased cell proliferation as measured by MTT assay. Four days after cell seeding, the numbers of U251n-hpa cells were significantly increased as compared to U251n-pc cells. **, *P *< 0.01, compared with U251n-pc cells. (B) U251n-hpa cells developed more and bigger colonies than U251n-pc cells as measured by colony formation assay. **, *P *< 0.01, compared with U251n-pc cells.

To test the possibility of heparanase on cell growth, cell proliferation and anchorage-independent growth was therefore evaluated. Growth curve was measured during 4 days in culture. Initially 4 × 10^3 ^cells were plated in each well of a 96-well plate, and cell numbers were determined daily by MTT assay. Four days after seeding, the cell number of the U251-hpa group was significantly higher than that of the U251n-pc group (*P *< 0.01) (Figure 4A). Furthermore, the anchorage-independent cell growth was determined by colony formation in soft agar. U251n-hpa cells formed significantly higher numbers of colonies in soft agar and the colonies were generally bigger than those of control U251n-pc cells (Figure 4B).

### Heparanase overexpression induces phosphorylation of AKT

**Figure 5:**
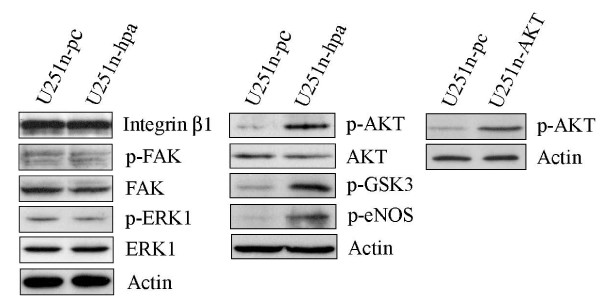
**Heparanase regulates signal pathways**. U251n-hpa cells showed increased phospho-AKT and AKT downstream targets, phospho-eNOS and phospho-GSK3. β1 integrin, phospho-FAK and phospho-ERK levels were not significantly changed.

To investigate the involvement of signaling cascades that might be activated by heparanase, the phosphorylation levels of AKT, ERK1, integrin-mediated FAK and β1 integrin were examined by Western blot analysis. The phosphorylation levels of AKT were noticeably increased in heparanase-overexpressing U251n cells. To confirm the change of p-AKT in heparanase overexpressing cells, Akt downstream targets, p-eNOS and p-GSK3, were determined by Western blot. Increased phosphorylstion of eNOS and GSK3 were found in correlation with the change of p-AKT in U251n-hpa cells. The β1 integrin, phosphorylation of FAK and ERK1 appeared unchanged (Figure 5).

## Discussion

In addition to proteolytic enzymes like MMPs and serine proteases, heparanase, given its ability to degrade HSPGs, may play a role in cancer cell invasion. Using U251n cells, we provide evidence that enhanced heparanase significantly increases cell invasion. The increased cell invasion can be significantly blocked by heparin, a heparanase inhibitor [28-31], indicating that cell invasion is a direct effect of heparanase enzyme activity. Apart from its traditional function, heparanase shows a potential in regulating cell chemotactic migration, cell proliferation and anchorage-independent colony formation which seems to be independent of the degradation of ECM. Heparanase may involve in regulation of gene expression through its enzyme activity. Like what have been found in this work, increased AKT phosphorylation might be one of the targets of heparanase regulation. We have no direct evidences to conclude that increased cell migration and growth by heparanase-overexpression is the results of elevated AKT phosphorylation. As AKT is involved in multiple cellular functions [32], the increased AKT phosphorylation may contribute to the changes of these cell function.

In fact, heparanase has been reported in the regulation of various signal transduction pathways [19,21,22,33]. However, the transduction pathway likely differs in different cell types. For glioma cells, overexpression of heparanase in U87 cells causes increased phosphorylation of FAK and AKT, decreased phosphorylation of ERK and unchanged phosphorylation of p38 [20]. In another study, overexpression of heparanase in rat C6 glioma cells shows increased phosphorylation of p38, but phosphorylation of ERK remain unchanged [21]. Our results demonstrate that overexpression of heparanase in U251n cells increases phosphorylation of AKT, while phosphorylation of ERK1 and FAK is not altered. Thus, heparanase may affect several key signaling components essential for tumor progression. A key issue is to determine the heparanase binding site and how heparanase regulates gene expression and various signaling proteins.

In summary, using well described human glioma cell line U251n [34,35], we found that heparanase has the potential to regulate tumor cell invasion, chemotactic migration and proliferation. AKT signaling might be the target of heparanase. Further studies on different tumor cell lines are warranted.

## Competing interests

The authors declare that they have no competing interests.

## Authors' contributions

XH conceived of the study and wrote the manuscript. SN, HJ and FJ participated in the design of the study and helped write the paper. ZGZ, TM and MC participated in the design of the study and reviewed manuscript. XPZ and XGZ carried out the gene transfection. All authors read and approved the final manuscript.
